# Adolescent traumatic brain injury leads to incremental neural impairment in middle-aged mice: role of persistent oxidative stress and neuroinflammation

**DOI:** 10.3389/fnins.2023.1292014

**Published:** 2023-10-25

**Authors:** Ziyuan Chen, Pengfei Wang, Hao Cheng, Ning Wang, Mingzhe Wu, Ziwei Wang, Zhi Wang, Wenwen Dong, Dawei Guan, Linlin Wang, Rui Zhao

**Affiliations:** ^1^Department of Forensic Pathology, School of Forensic Medicine, China Medical University, Shenyang, Liaoning, China; ^2^Key Laboratory of Environmental Stress and Chronic Disease Control and Prevention, Ministry of Education, China Medical University, Shenyang, Liaoning, China; ^3^Liaoning Province Key Laboratory of Forensic Bio-Evidence Sciences, Shenyang, China

**Keywords:** traumatic brain injury, anxiety and depression, cognitive dysfunction, neuroinflammation, oxidative stress

## Abstract

**Background:**

Traumatic brain injury (TBI) increases the risk of mental disorders and neurodegenerative diseases in the chronic phase. However, there is limited neuropathological or molecular data on the long-term neural dysfunction and its potential mechanism following adolescent TBI.

**Methods:**

A total of 160 male mice aged 8 weeks were used to mimic moderate TBI by controlled cortical impact. At 1, 3, 6 and 12 months post-injury (mpi), different neurological functions were evaluated by elevated plus maze, forced swimming test, sucrose preference test and Morris water maze. The levels of oxidative stress, antioxidant response, reactive astrocytes and microglia, and expression of inflammatory cytokines were subsequently assessed in the ipsilateral hippocampus, followed by neuronal apoptosis detection. Additionally, the morphological complexity of hippocampal astrocytes was evaluated by Sholl analysis.

**Results:**

The adolescent mice exhibited persistent and incremental deficits in memory and anxiety-like behavior after TBI, which were sharply exacerbated at 12 mpi. Depression-like behaviors were observed in TBI mice at 6 mpi and 12 mpi. Compared with the age-matched control mice, apoptotic neurons were observed in the ipsilateral hippocampus during the chronic phase of TBI, which were accompanied by enhanced oxidative stress, and expression of inflammatory cytokines (IL-1β and TNF-α). Moreover, the reactive astrogliosis and microgliosis in the ipsilateral hippocampus were observed in the late phase of TBI, especially at 12 mpi.

**Conclusion:**

Adolescent TBI leads to incremental cognitive dysfunction, and depression- and anxiety-like behaviors in middle-aged mice. The chronic persistent neuroinflammation and oxidative stress account for the neuronal loss and neural dysfunction in the ipsilateral hippocampus. Our results provide evidence for the pathogenesis of chronic neural damage following TBI and shed new light on the treatment of TBI-induced late-phase neurological dysfunction.

## Introduction

Traumatic brain injury (TBI) is the leading cause of disability and death in both children and young adults (Maas et al., 2017; Quaglio et al., 2017). Accumulated evidence in the past decades demonstrates that young adults and adolescent survivors after TBI may present cognitive, behavioral, or mental symptoms in their adulthood or late life (Moretti et al., 2012; Richmond and Rogol, 2014; Sun et al., 2017; Rodgin et al., 2021; Xu et al., 2021; Bourke et al., 2022; Max et al., 2022; Sanchez et al., 2022). Thereafter, TBI has been commonly recognized as a risk factor for many neurodegenerative diseases, such as Alzheimer’s disease, Parkinson’s disease, dementia, and chronic traumatic encephalopathy (VanItallie, 2019; Vázquez-Rosa et al., 2020; Brett et al., 2022). However, the underlying mechanisms of late-life neural dysfunction post TBI remains unclear.

Both primary and secondary injuries contribute to neuronal damage in the acute and subacute phase of TBI. Intracranial hemorrhage and brain destruction are caused by the initial external force (Maegele et al., 2017), followed by the reactive microglia and astrocytes that alter their transcriptional and morphological profiles and exert pro-or anti-inflammatory effects (Karve et al., 2016), which promotes the clearance of damaged tissue and neural regeneration after TBI (Corps et al., 2015). In the chronic phase, damage-associated molecular patterns, such as excitotoxicity, mitochondrial dysfunction, oxidative stress, and neuroinflammation, trigger secondary injuries (Braun et al., 2017). Chronic neuroinflammation, supported by the persisting reactive astrocytes and microglia in the injured animal and human brain (Smith et al., 1997; Ramlackhansingh et al., 2011; Faden et al., 2016; Pischiutta et al., 2018; van Vliet et al., 2020), can last for an extended period and contribute to the chronic neurological disorder post-injury (Faden et al., 2016; van Vliet et al., 2020). In addition, oxidative stress has been well recognized as a common denominator of both brain injury and TBI-related neurodegenerative disease (Mackay et al., 2006; Ma et al., 2017; Dong et al., 2018; Cheng et al., 2022). Excessive reactive oxygen species (ROS) and reactive nitrogen species are generated and cause neurotoxicity by increasing intracellular free Ca^2+^ and releasing excitatory amino acids (Khatri et al., 2021) or directly inducing peroxidation of lipid, protein, and DNA in the acute phase of TBI (Ma et al., 2017). These findings provide evidence to link chronic inflammation and oxidative stress with TBI-related neurodegenerative pathology.

Nuclear factor erythroid-derived 2-related factor 2 (NRF2), is widely recognized as a key transcription factor that regulates both the antioxidant responses and neuroinflammation (Buendia et al., 2016). Our previous study presented that NRF2 is widely expressed in neurons and glia post TBI (Dong et al., 2019). Many evidence has demonstrated that NRF2 plays neuroprotective roles against TBI-induced brain injury (Li et al., 2014; Lu et al., 2015; Dong et al., 2018; Sigfridsson et al., 2020; Wang et al., 2020; Cui et al., 2021) and neurodegenerative diseases (Rojo et al., 2010, 2018; Osama et al., 2020; Ren et al., 2020; Zgorzynska et al., 2021). Considering the critical role of NRF2 in TBI and numerous neurodegenerative diseases, it is necessary to explore the expression pattern of NRF2 during the late phase of TBI.

In this study, to reveal the underlying mechanisms of long-lasting neurological dysfunction following TBI in mice, we explored the lifespan change of neurological dysfunction, oxidative stress and neuroinflammation in ipsilateral hippocampus from young age to late life of TBI mice.

## Materials and methods

### Animals and controlled cortical impact models

A total of 160 male C57BL/6 mice (8 weeks old, 20–26 g) were used in our study. Mice were randomly divided into TBI and age-matched control groups. Then mice were subdivided into four subgroups as the indicated timepoints (1, 3, 6 and 12 mpi, *n* = 20 in each subgroup). All the mice were housed under constant temperature (23 ± 1°C), humidity (60%) and a 12-h light–dark cycle, with free access to food and water. In the TBI group, CCI was used to mimic the stable moderate TBI model (considerable cortical tissue loss without hippocampal injury) as in our previous studies (Siebold et al., 2018; Ma et al., 2019; Cheng et al., 2023). Briefly, the mice were placed on a stereotaxic apparatus after being anesthetized. A scalp incision was subsequently made at the midline to expose the skull. A 4 mm craniotomy was performed in the left hemisphere between the bregma and lambda to expose the dura mater. The craniocerebral strike apparatus (PinPoint™ PCI3000, Hatteras instruments, America) was used to perform a vertical impact on the cortex (3 mm diameter impactor, velocity 1.5 m/s, residence time 50 ms, depth 1 mm), followed by sutured scalped. The comatose mice were then placed on a 37°C heating pad and returned to the cages until the vital signs were stable. The mice in the sham group underwent the same surgical procedure without any cortical impact. No mice died after the surgery or during feeding. All the experiments were approved by the Animal Ethics Committee of China Medical University.

### Behavior tests

The following behavioral tests were conducted at the indicated timepoints post injury. Mice were tested by sucrose preference test (SPT) and elevated plus maze (EPM) first, and followed by the force swimming test (FST) and Morris water maze (MWM). To avoid the effects among different behavior tests, mice were kept in the cages for 1 day to recover before the next test.

#### SPT

The SPT was adapted from a previous protocol (Leng et al., 2018; Browne et al., 2022). Briefly, each mouse was allowed free access to 1% sucrose solution and tap water for 16 h, and the placement of the two bottles was changed every 12 h to avoid side preference at the training stage. After 12 h of food deprivation, the mice were allowed free access to tap water and 1% sucrose solution for the next 24 h. Sucrose preference was calculated as the percentage of the consumed sucrose solution from the total consumed fluid (sucrose + water).

#### FST

The FST was performed as previously described (Li et al., 2021). Briefly, the mice were placed in a cylindrical glass filled with no less than three quarters of water for 6 min, and their activity was recorded for the last 5 min at a temperature of 23 ± 1°C. SMART™ tracking software (San Diego Instruments, San Diego, CA, USA) was used to record the time of immobility.

#### EPM

To measure anxiety-like behavior post-injury, the EPM test was performed as previously described (Toshkezi et al., 2018). The EPM was consisted of two opened arms and two closed arms. Mice were placed at the center of the junction of the maze facing one open arm and allowed to explore freely for 5 min. The spent time and number of entries into the open arms were recorded using SMART™ tracking software for further analysis.

#### MWM

To evaluate the spatial memory of mice post injury, we performed the MWM test at 1, 3, 6 and 12 mpi as previously described (Ren et al., 2020). Briefly, the mice were trained to find a platform that relied on distal cues from four different starting positions in an open circular tank. Mice that failed to find the platform within 60 s were guided and remained on the platform for 30 s before being returned to their cages. On the first day, mice were trained to find the platform 0.5 cm above the water. Then, the mice were trained for four more days to find the platform below the opaque water. On the sixth day, mice were placed at the starting quadrant opposite to the quadrant with removed platform and several variables were recorded and measured for 60 s: the distance in the platform quadrant, time spent in the platform quadrant, and number of crossings between the platform and platform quadrant, using SMART™ tracking software (San Diego Instruments, San Diego, CA, United States).

### Animal selection and sample collection

TBI mice with abnormal behavior were selected for the subsequent biological experiments (*n* ≥ 12 in each time point) according to the method proposed by Rodney M Ritzel with a slight modification (Ritzel et al., 2022). Briefly, we established a baseline according to the means of behavioral parameters (SPT, EPM, MWM and FST) from age-matched control mice, and then evaluate the neurological dysfunction of TBI mice. The mice with behavioral parameters exceeding the baseline were selected for the followed experiments. The proportion of TBI mice meeting these criteria was presented in Supplementary Table S3. Brain samples were collected as previously described (Dong et al., 2018). For immunoblotting, malondialdehyde (MDA) detection and quantitative real time polymerase chain reaction (RT-qPCR), mice were perfused with cold phosphate buffered saline. The ipsilateral hippocampus was dissected on ice and placed in liquid nitrogen for use. For immunohistochemical or immunofluorescence staining, the mice were perfused with cold 4% paraformaldehyde. The brains were embedded in paraffin and 5 μm sections were prepared for histological staining.

#### MDA

MDA was detected using the commercial kit (Nanjing Jiancheng Bioengineering Institute, Nanjing, China). The total protein concentration was quantified with the bicinchoninic acid assay kit (Beyotime, P0009) according to the manufacturer’s instructions. The MDA content was expressed in nmol/mg protein.

#### Immunofluorescence and immunohistochemistry

Immunofluorescence and immunohistochemical staining were performed as previously described (Dong et al., 2018). Breifly, tissue sections were deparaffinized and hydrated, followed by antigen retrieval in citrate buffer at 95°C for 5 min. Then sections were incubated by primary and secondary antibodies, which were listed in the Supplementary Table S1. Three animals per group were used for immunofluorescence or immunohistochemical staining, and the definite fields of two sections per animal were evaluated (*n* = 6). The number of positive cells in the ipsilateral hippocampal CA1 and hilus was counted at ×200 magnification. The average optical density and positive area were quantified using ImageJ, version 6.0 (National Institutes of Health).

#### Fluoro-Jade C staining

Fluoro-Jade C (FJC; Millipore, AG325–30MG) was used to assess the number of degenerating neurons following injury. As previously described (Dong et al., 2018), the sections of ipsilateral hippocampus were incubated with 0.06% potassium permanganate solution and then incubated in 0.01% FJC solution for 10 min.

#### Western blot

The ipsilateral hippocampus was collected for protein extraction as previously described (Dong et al., 2018). Protein concentration was measured using a BCA kit (P0012; Beyotime Biotechnology, Shanghai, China). The primary and secondary antibodies used in this study are listed in Supplementary Table S1. Relative band density was quantified using ImageJ software (National Institutes of Health).

#### RT-qPCR

RNA extraction, and RT-qPCR were performed as previously described (Dong et al., 2018). The housekeeping gene *Gapdh* was amplified to ensure the addition of equal amounts of cDNA to the PCR reactions. ΔΔCT was used to quantify the target gene expression, with *Gapdh* as the reference gene. Primers used in this study were listed in Supplementary Table S2.

### Morphological data collection and Sholl analysis

Fluorescence images were captured using Zeiss Axio Scan.Z1 under ×200 magnification. Morphological reconstruction of glial fibrillary acidic protein (GFAP) positive astrocytes was performed using ImageJ (version 6.0; National Institutes of Health), as previously described (Bondi et al., 2021). Briefly, images of the ipsilateral hippocampal hilus were converted to an 8-bit format. Two sections were taken from each animal, and two GFAP-positive cells (*n* = 12) were randomly selected from the hilus region of each section. Then the total and maximum lengths of the process and the number of intersections were measured with circles of different diameters increasing by 4 μm from the center of the cell body.

### Statistical analysis

Data are expressed as mean ± standard deviation. All the data were analyzed using Prism software (version 8.0; GraphPad Software, Inc.). Two-way ANOVA was used to measure the differences between Sham and TBI groups of different time points, and multi-group comparisons were performed using Tukey’s *post hoc* test. Mann–Whitney U test was used for comparison between two groups with non-normal distributed data. Statistical significance was set at *p* < 0.05.

## Results

### Adolescent TBI causes exacerbated neural impairment in the middle-aged mice

**Figure 1:**
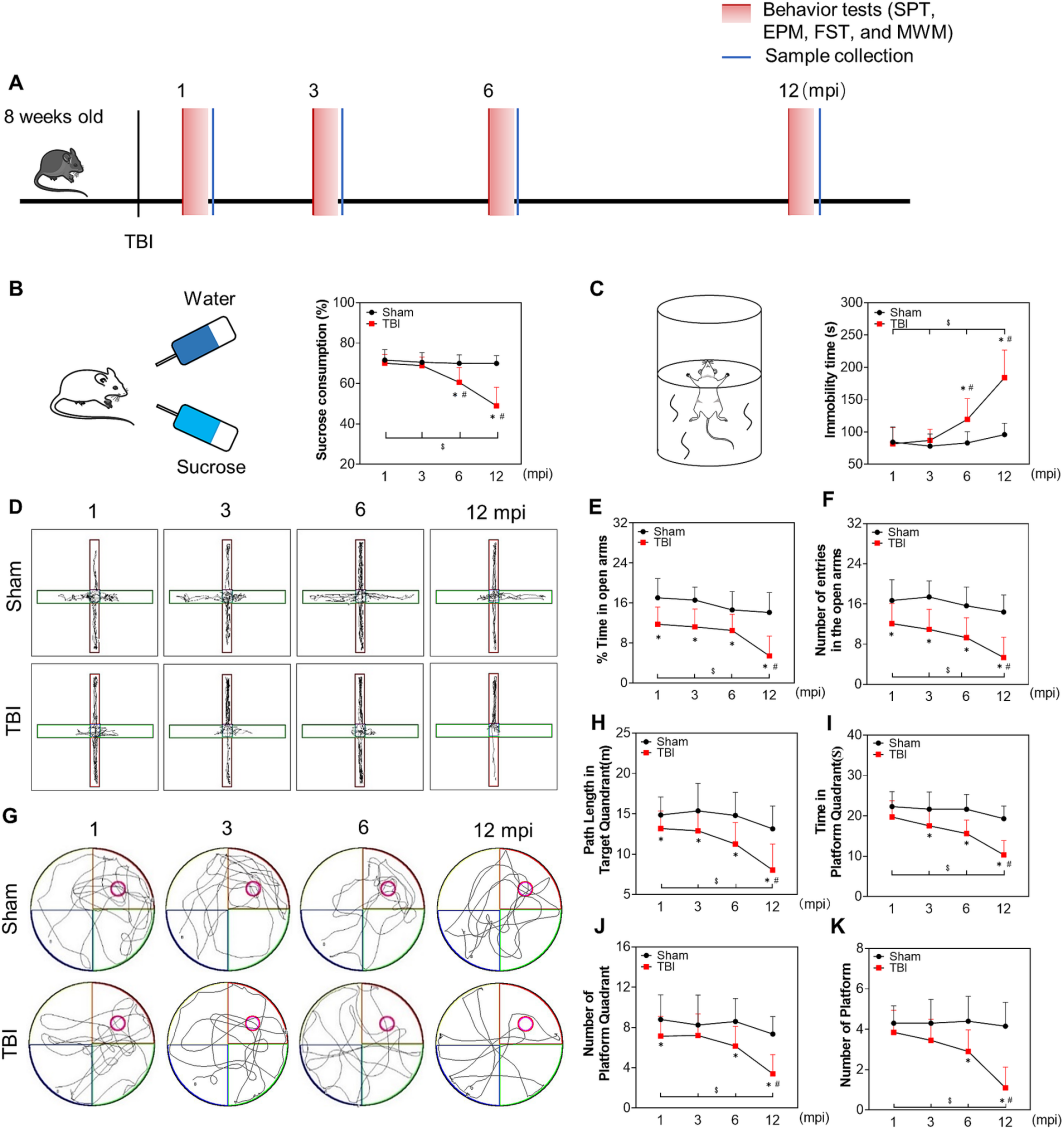
The late phase changes on neurological function post injury. **(A)** Schematic diagram of the experimental design. **(B)** Schematic diagram of the SPT (left panel) and the percentage of sucrose consumption over total fluid volume of mice at 1, 3, 6 and 12 mpi (right panel). **(C)** Schematic diagram of the FST (left panel) and the statistical analysis of immobility time at each indicated timepoints (right panel). **(D)** The representative moving path of mice in EPM test at 1, 3, 6 and 12 mpi. **(E)** The percentage of time in the open arm. **(F)** The number of entries in the open arm. **(G)** The representative moving path of MWM in probe test at 1, 3, 6 and 12 mpi. **(H)** The path length in the platform quadrant. **(I)** The time in the platform quadrant. **(J)** The number of platform quadrant. **(K)** The number of platform. *n* = 20, *p < 0.05 compared with the age-matched sham (Mann–Whitney U Test); $*p* < 0.05 1, 3, 6 mpi versus 12 mpi (Tukey’s *post hoc* test); #*p* < 0.05 compared with the preceding adjacent TBI group (Tukey’s *post hoc* test).

To explore neural function impairment during the late phase of TBI, we conducted the SPT, FST, EPM, and MWM to evaluate depression-like and anxiety-like behaviors and memory deficits at 1, 3, 6 and 12 mpi (Figure 1A). As shown in Figures 1B,C, the mice in the TBI group demonstrated a significant decrease in the sucrose consumption and increase in the immobility time compared with sham mice from 6 mpi, which was exacerbated sharply at 12 mpi, suggesting that injured mice exhibited depression-like behaviors at 6 mpi and beyond. Furthermore, the decrease of the number and the time of entries in the open arms were worsened at 12 mpi in EPM (Figures 1D–F), demonstrating a prolonged anxiety-like behavior in injured mice. In MWM test (Figure 1G), the path length, the time in the platform quadrant (Figures 1H,I), the number of platform quadrants (Figure 1J) and the number of platform in the probe test (Figure 1K) decreased in the TBI mice compared with the age-matched mice; all of them were significantly decreased at 12 mpi (Figures 1H–K), indicating a persistent and incremental spatial memory impairment post-injury. In summary, these behavioral observations revealed that TBI in adolescent mice resulted in the deterioration of neurological function in the late phase of their life (6 mpi and 12 mpi).

### Apoptosis contributes to chronic neuron loss in ipsilateral hippocampus following TBI

**Figure 2:**
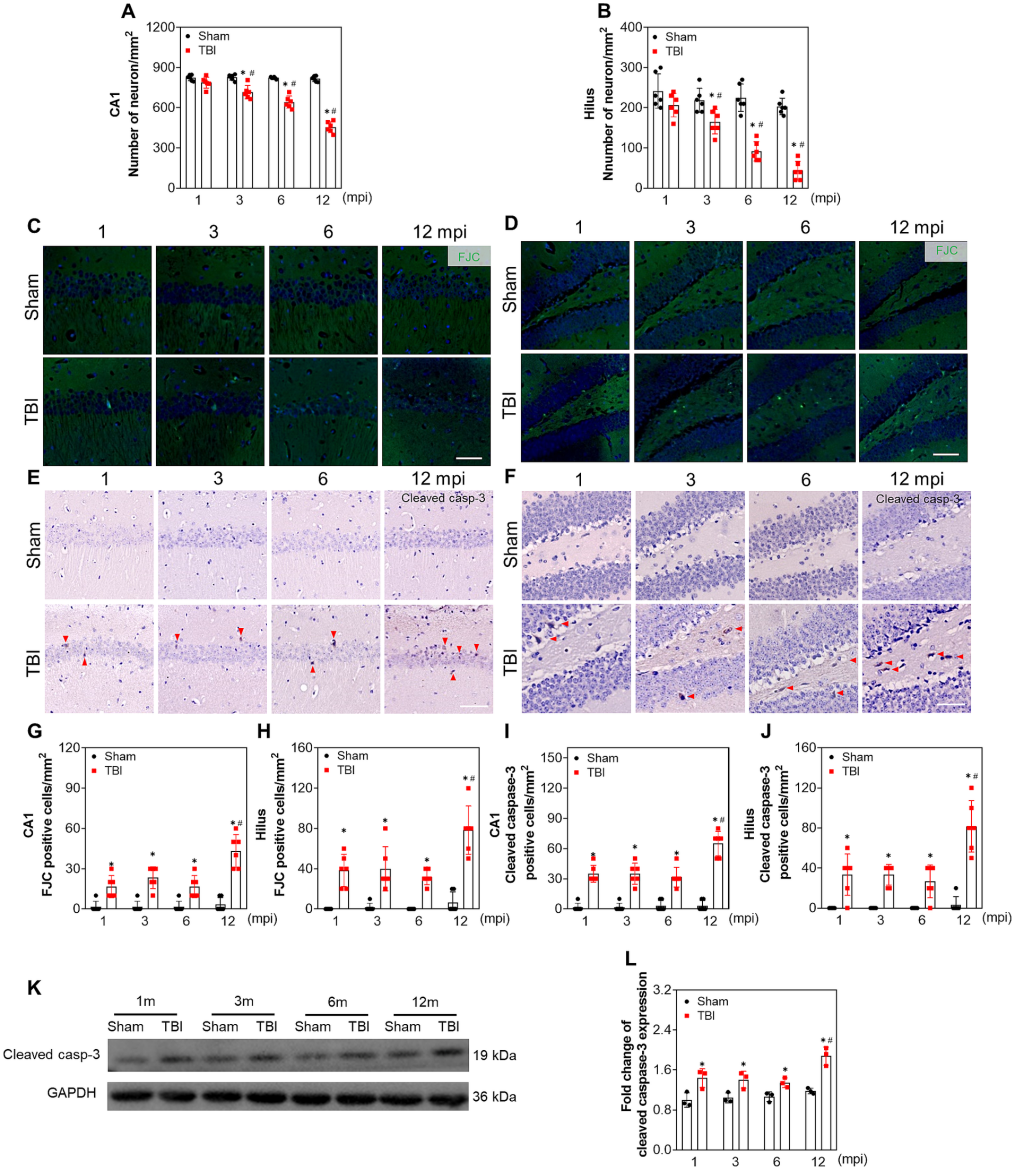
Apoptosis contributes to chronic neuron loss in ipsilateral hippocampus following TBI. **(A,B)** The quantitative analysis of the number of NeuN positive cells in CA1 and hilus at different indicated time points post injury, *n* = 6. **(C,D)** Representative images of FJC staining in CA1 and hilus of ipsilateral hippocampus at different time post injury. **(E,F)** Representative immunostaining images of cleaved caspase-3 in CA1 and hilus of ipsilateral hippocampus at different time post injury. **(G–J)** The quantitative analysis of the number of FJC and cleaved caspase-3 positive cells in CA1 and hilus at different time post injury, *n* = 6. **(K,L)** The representative immunoblots and relative densities of cleaved caspase-3 in the ipsilateral hippocampus of each group; *n* = 3. Scale bar, 20 μm. *p < 0.05 compared with the age-matched sham mice (Mann–Whitney U Test); #*p* < 0.05 compared with the TBI mice of preceding adjacent group (Tukey’s *post hoc* test).

Since the hippocampal neurons play the key roles of both processing memory storage and regulating depression/anxiety-like behaviors (Tunc-Ozcan et al., 2019; Takahashi et al., 2021), we explored the relationship between neural dysfunction and ipsilateral hippocampal neuron loss. A progressive neuronal decrease in ipsilateral hippocampal CA1 (Figure 2A; Supplementary Figure S1) and hilus (Figure 2B; Supplementary Figure S1) was revealed by the NeuN-positive staining from 3 mpi, which was not affected by the ageing of the mice (Figures 2A,B). In addition, as shown in Figures 2C,D,G,H, FJC-positive cells remained at a constant low level within 6 months post-injury and increased sharply at 12 mpi both in CA1 and hilus. In line with this, immunohistochemical staining of cleaved caspase 3 demonstrated the same pattern (Figures 2E,F,I,J). Although the objective number of apoptotic cells gradually increased with ageing, no statistical difference was observed among the mice with different age in the sham group (Figures 2G–J), demonstrating ageing-related neuron apoptosis in the hippocampus could not account for the neural dysfunction in middle-aged mice. Furthermore, the protein band of cleaved-caspase 3 in the TBI hippocampus was densified at 12 mpi compared with the age-matched mice (Figures 2K,L), which accounted for the exacerbated neural dysfunction in the middle-aged mice. Our data revealed that neuronal apoptosis in the ipsilateral hippocampus contributes to chronic neural dysfunction following TBI.

### Chronic persistent oxidative stress is associated with robust neuronal damage in middle-aged mice

**Figure 3:**
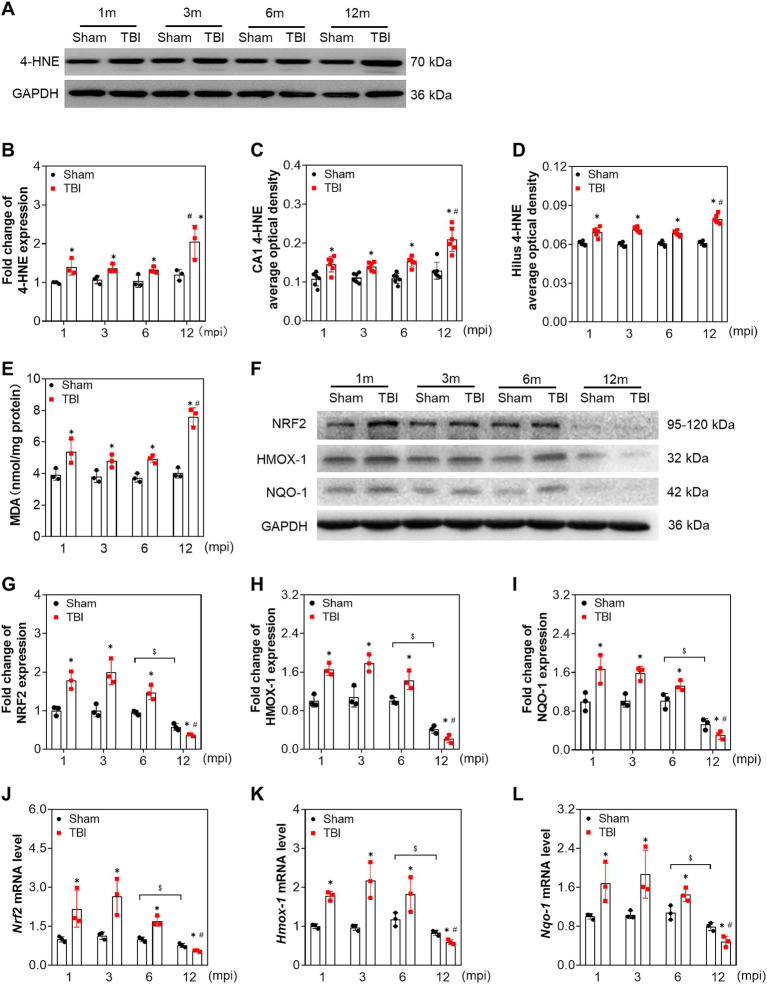
Adolescent TBI-induced persistent oxidative stress is enhanced in middle-aged mice. **(A,B)** Representative immunoblots and relative densities of 4-HNE in the ipsilateral hippocampus at 1, 3, 6 and 12 mpi, *n* = 3. **(C,D)** The quantitative analysis of the average optical density of 4-HNE immunohistochemical staining in CA1 and Hilus, *n* = 6. **(E)** The quantitative analysis of MDA content, *n* = 3. **(F)** Representative immunoblots of NRF2, HMOX-1, NQO-1 of the ipsilateral hippocampus at 1, 3, 6 and 12 mpi. **(G–I)** Relative densities of NRF2, HMOX-1, NQO-1, *n* = 3. **(J–L)** The mRNA levels *Nrf2*, *Hmox-1*, *Nqo-1* of the ipsilateral hippocampus at 1, 3, 6 and 12 mpi, *n* = 3. *p < 0.05 compared with age-matched sham (Mann–Whitney U Test); $*p* < 0.05 comparison between sham groups (Tukey’s *post hoc* test); #*p* < 0.05 compared with the TBI mice of preceding adjacent group (Tukey’s *post hoc* test).

To explore whether neuronal damage is associated with chronic oxidative stress during the pathological process following TBI, we first evaluated the protein level of 4-hydroxynonenal (4-HNE), a marker of lipid peroxidation. As shown in Figures 3A,B, the level of 4-HNE in the TBI groups was elevated compared with the corresponding sham mice at all indicated timepoints, which was increased sharply at 12 mpi (fold change = 2.05 ± 0.43). Then, 4-HNE was further confirmed by immunostaining, and the average optical density of 4-HNE in the CA1 and hilus increased following injury (Figures 3C,D; Supplementary Figures S2A,B). To further evaluate oxidative stress alteration, MDA was detected in the ipsilateral hippocampus. As shown in Figure 3E, MDA was elevated with the same trend as 4-HNE at all intervals post-injury. Unexpectedly, no difference was observed on 4-HNE and MDA in the hippocampus of the sham mice (Figures 3B–E). To assess endogenous protective capability, we subsequently determined the level of the antioxidant response. NRF2 and its regulated antioxidant enzymes, heme oxygenase-1 (HMOX-1) and quinone oxidoreductase-1(NQO-1), were upregulated at both protein and mRNA levels following TBI within 6 mpi (Figures 3F–L) and significantly decreased at 12 mpi both in the TBI and its age-matched mice (Figures 3F–L). Whereas, ccompared with the age-matched sham mice, the protein level of glutamate-cysteine ligase catalytic subunit (GCLC) and its modifier subunit (GCLM) were only decreased at 12 mpi, with no significant changes at 1, 3, and 6 mpi (Supplementary Figures S3A–D). Our data indicated that persistent oxidative stress contributes to neuronal damage following TBI, which was also related to the ageing.

### Persistent inflammation induced by TBI is exacerbated in middle-aged mice

**Figure 4:**
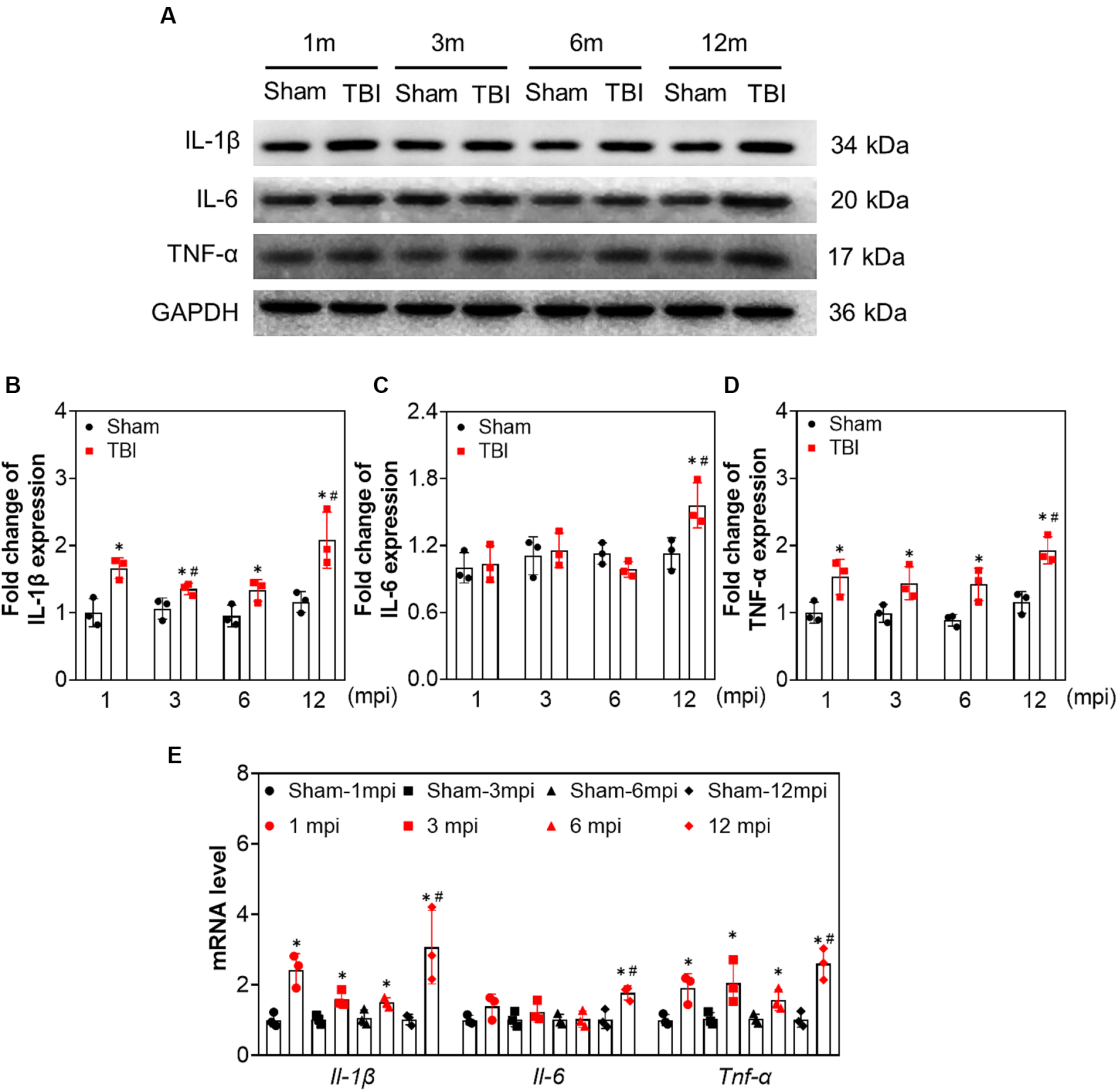
The increased expression of proinflammatory cytokines in ipsilateral hippocampus induced by adolescent TBI. **(A–D)** Representative immunoblots and relative densities of IL-1β, IL-6 and TNF-α of the ipsilateral hippocampus, *n* = 3. **(E)** The mRNA levels of *Il-1β, Il-6* and *Tnf-α* of the ipsilateral hippocampus, *n* = 3. *p < 0.05 compared age-matched sham (Mann–Whitney U Test); #*p* < 0.05 compared with the TBI mice of preceding adjacent group (Tukey’s *post hoc* test).

To explore the changes in neuroinflammation in the ipsilateral hippocampus at different intervals post-injury, we tested the protein and mRNA levels of interleukin (IL)-1β, IL-6, and tumor necrosis factor-alpha (TNF-α). As shown in Figure 4, the protein and mRNA levels of IL-1β and TNF-α were elevated following TBI compared with the age-matched sham mice at each indicated time point. However, IL-6 expression was only increased at 12 mpi (fold change = 1.56 ± 0.20) and revealed no significant difference at 1, 3, and 6 mpi (Figures 4B–E). Our data demonstrated that long-lasting neuroinflammation in the ipsilateral hippocampus was present following TBI, which was aggravated in middle-aged mice (12 mpi).

### Alteration of hippocampal astrocytes during the chronic phase of TBI

**Figure 5:**
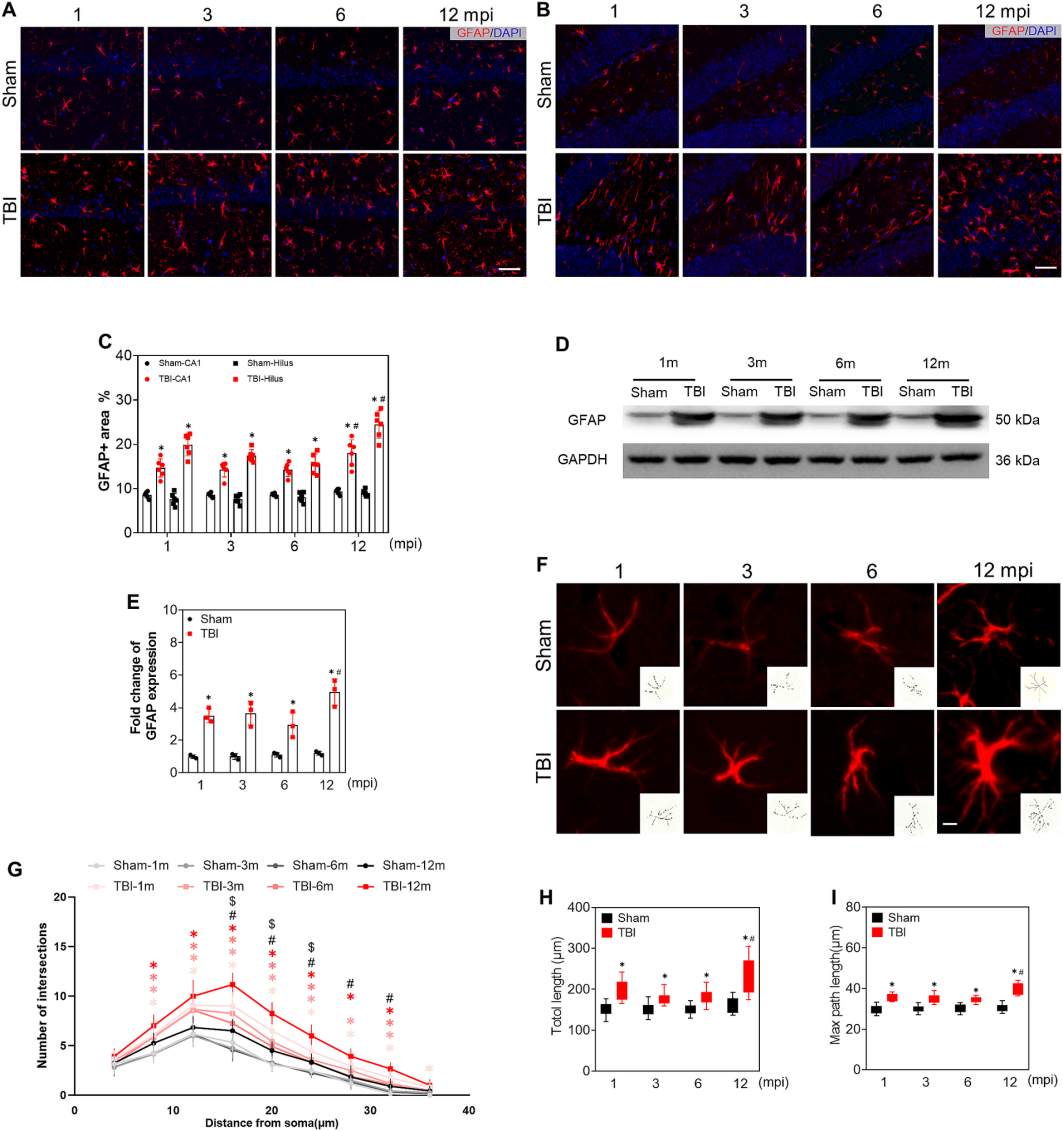
The alteration of reactive astrocytes during the chronic phase of adolescent TBI. **(A,B)** Representative images of GFAP (red) immunofluorescent staining of CA1 and hilus of ipsilateral hippocampus; scale bar, 20 μm; *n* = 6. **(C)** The quantitative analysis of GFAP positive area of CA1 and hilus, *n* = 6. **(D,E)** Representative immunoblots and relative densities of GFAP of the ipsilateral hippocampus at 1, 3, 6 and 12 mpi, *n* = 3. **(F)** Representative image of GFAP+ astrocytes in hilus at 1, 3, 6 and 12 mpi, the reconstructed skeleton of representative astrocytes was present in the lower right corner of the image; scale bar, 5 μm. **(G)** Intersection profile of astrocytes in hilus at 1, 3, 6 and 12 mpi, *n* = 12. **(H)** The quantitative analysis of total length of astrocytic process. **(I)** The quantitative analysis of the max length of astrocytic process; *n* = 12. *p < 0.05 comparison between TBI and age-matched Sham groups (Mann–Whitney U Test); #*p* < 0.05 comparison between 6 and 12 mpi groups (Tukey’s *post hoc* test); $*p* < 0.05, comparison between Sham mice of 6 and 12 mpi groups (Tukey’s *post hoc* test).

To assess the reactive astroglia, we detected GFAP expression by immunofluorescence, a marker of astrocytes. As shown in Figures 5A,B, the immunoreactivity of GFAP and the percentage of GFAP+ area in the ipsilateral hippocampus were higher than those in sham mice at each time point, especially at 12 mpi (Figure 5C). In line with these, the protein levels of GFAP were also upregulated upon Western blot (Figures 5D,E). Our data proved that TBI led to astrogliosis in the hippocampus, and the astrocytic response was intensified in the hippocampus of middle-aged mice. It has been known that A1 astrocytes contribute to the pathogenesis of TBI and neurodegenerative diseases (Diaz-Castro et al., 2019; Song et al., 2020; Taylor et al., 2020). We explored the A1 astrocytes by C3d and GFAP double immunostaining. Several C3d + astrocytes signal colocalized with GFAP+ cells in the ipsilateral hippocampus post injury (Supplementary Figure S4A, lower panel). Whereas, no double-positive astrocyte was found in the hippocampus of sham mice from adolescence to middle age (Supplementary Figures S4A,B). Then the complexity of the reactive astrocytes was evaluated in the ipsilateral hippocampus by Sholl analysis. The representative astrocytes and their reconstructed skeleton in each group were shown in Figure 5F. A significant difference in the morphological complexity of astrocytes in sham mice was observed only in middle-aged mice (12 mpi) (Figure 5G). However, in TBI mice, the reactive astrocytes sustained the complex morphology post injury, which was presented by the total and maximum process length in Figures 5H,I. Our data revealed the alternation of reactive astrocytes and their morphological complexity in the ipsilateral hippocampus after TBI.

### Reactive microglia is aggravated in the late phase of TBI

**Figure 6:**
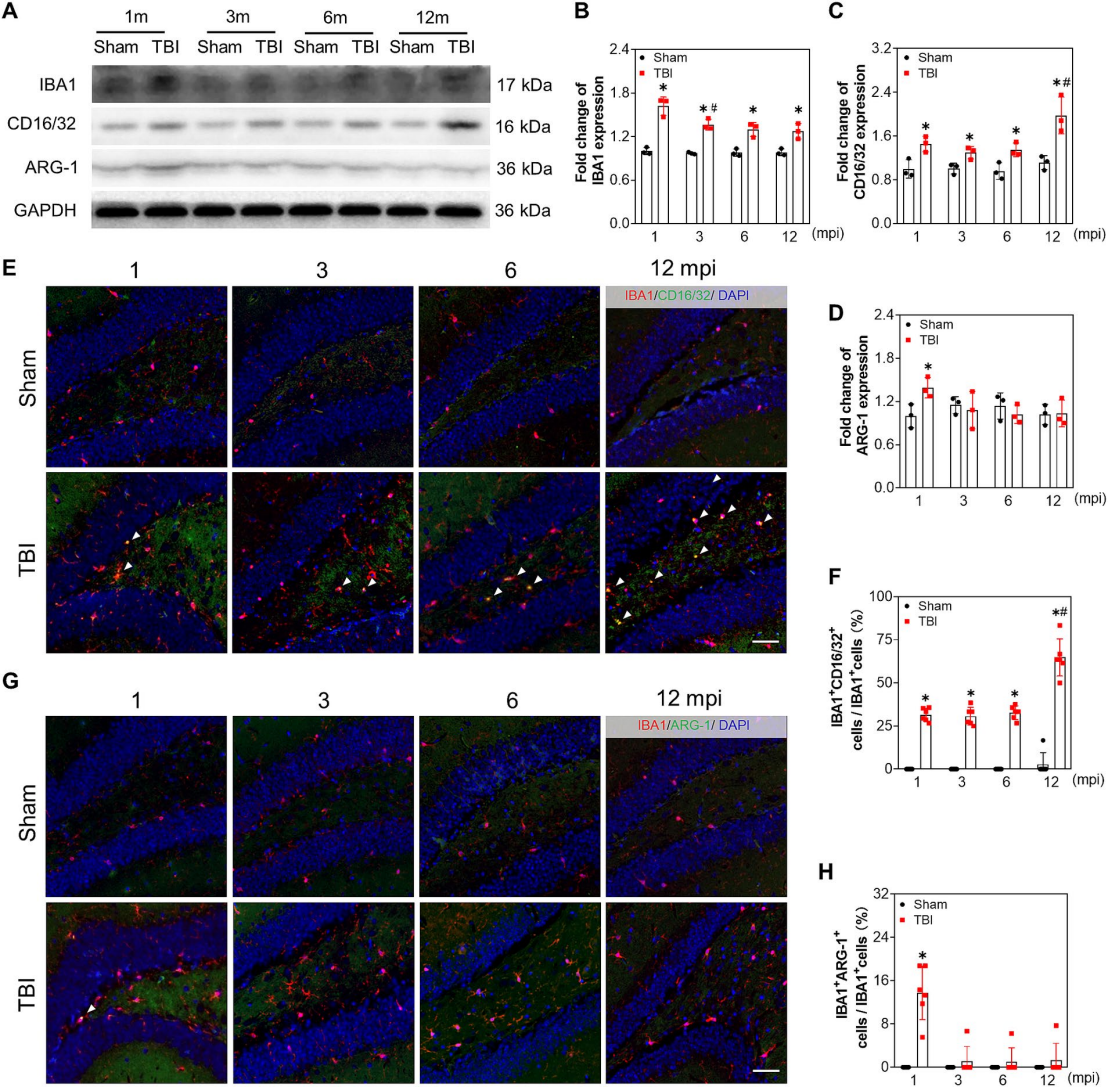
Reactive microglia is aggravated in the late phase of adolescent TBI. **(A–D)** Representative immunoblots and relative densities of IBA1, CD16/32, ARG-1 of the ipsilateral hippocampus at 1, 3, 6 and 12 mpi, *n* = 3. **(E)** Representative images of immunofluorescent double staining of IBA1 (red) and CD16/32 (green) of the ipsilateral hippocampal hilus; white arrows, IBA1 and CD16/32 double positive cells. **(F)** The quantitative analysis of the percentage of IBA1 and CD16/32 double positive cells, *n* = 6. **(G)** Representative images of immunofluorescent double staining of IBA1 (red) and ARG-1 (green) of the ipsilateral hippocampal hilus; white arrows, IBA1 and ARG-1 double positive cells. **(H)** The quantitative analysis of the percentage of IBA1 and ARG-1 double positive cells, *n* = 6. *p < 0.05 compared with age-matched sham (Mann–Whitney U Test), #*p* < 0.05 compared with the TBI mice of preceding adjacent group (Tukey’s *post hoc* test); scale bar, 20 μm.

To evaluate the reactive microgliosis during the chronic phase of TBI, we measured the protein levels of ionized calcium binding adaptor molecule 1 (IBA1), CD16/32, and Arginase 1 (ARG-1). As shown in Figures 6A–C, the protein levels of IBA1 and CD16/32 were increased in the hippocampus of TBI mice at each indicated time point compared with that of sham mice. However, the ARG-1 level only increased slightly at 1 mpi in TBI mice (Figures 6A,D). Moreover, double immunofluorescence staining of IBA1 + CD16/32 and IBA1 + ARG-1 (Figures 6E,G) supported M1 polarization of microglia in TBI mice. The ratio of CD16/32+ microglia in the hilus at 1, 3, 6 mpi were 31.5 ± 3.8%, 30.7 ± 5.2% and 32.8 ± 3.9% separately, which increased sharply to 64.8 ± 10.8% at 12 mpi (Figure 6F). While ARG-1+ microglia accounted for approximately 13.7 ± 5.0% of the microglia at 1 mpi and 1.1 ± 2.7%, 1.0 ± 2.6%, 1.3 ± 3.1% at 3–12 mpi in hilus (Figure 6H). These data indicated that persistent reactive microglia could contributed to neuronal damage following TBI, especially in middle-aged mice.

## Discussion

**Figure 7:**
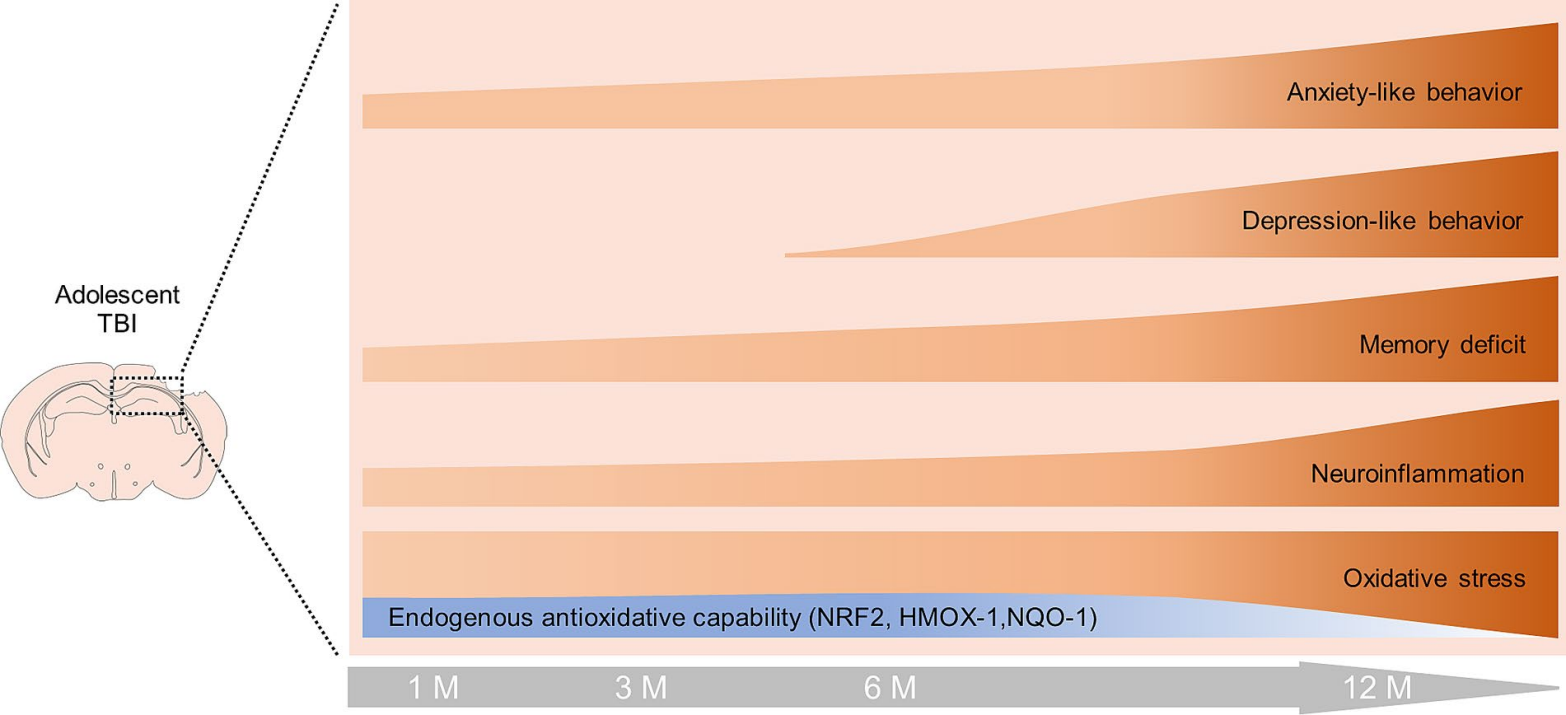
Schematic diagram of changes in neurological function, oxidative stress and neuroinflammation during the late phase of TBI. Oxidative stress and neuroinflammation occur in the hippocampus of adolescent TBI mice within 6 mpi, accompanied by increased expression of NRF2, HMOX-1, and NQO-1. TBI mice developed persistent memory impairment and anxiety behavior within 6 mpi, and began to develop depression-like behavior at 6 mpi. At 12 mpi, the expression of NRF2, HMOX-1, and NQO-1 was significantly decreased, leading to aggravated oxidative stress and neuroinflammation, as well as exacerbated memory impairment, anxiety-and depression-like behaviors.

Chronic pathophysiological changes post-TBI are responsible for neurological dysfunction and neurodegenerative diseases. Despite growing awareness of the chronic progressive nature of TBI (Tomaszczyk et al., 2014), there is still a need to characterize the pathogenesis of long-term neurological function post-injury. In this study, we described that adolescent TBI led to chronic neurological dysfunction, which was aggravated in middle-aged mice. Chronic oxidative stress, sustained reactive astrocyte and microgliosis contribute to the pathogenesis of neurological dysfunction and hippocampal neuron loss following TBI. Moreover, the aggravated neurological dysfunction in middle-aged TBI mice was partly due to the decrease of NRF2-mediated antioxidant response. Our present study suggests that ageing might be a contributory factor for long-term neural damage following TBI. The potential mechanism of long-term neural dysfunction after TBI is schematically illustrated in Figure 7.

Brain injuries trigger various neurological complications, including epilepsy, depression, and dementia (Burke et al., 2021; Stopa et al., 2021; Wang et al., 2021). Our observations revealed that adolescent traumatic brain injury results in spatial memory impairment and anxiety/depression-like behavior in middle-aged mice, which is consistent with the previous study (Mao et al., 2020). Although epidemiological study has revealed no obvious correlation between injury severity and depression occurrence (Vadlamani and Albrecht, 2020), previous study observed that mice underwent severe TBI showed depression-like behavior at 3 months post-injury (Mao et al., 2020), while this neural dysfunction was observed at 6 months post moderate TBI in our present study, the time delay of depression-like behavior post injury may be associated with the severity of TBI model.

In addition, local TBI could promote the secondary damage of certain remote brain regions in the late phase, resulting in a diverse range of neurological disorders. Numerous studies reported that chronic atrophy and degenerative disease of white matter post brain trauma aggravate cognitive dysfunction, depression, and apathy (Filley and Fields, 2016; Cole et al., 2018; Bartnik-Olson et al., 2021; Mohamed et al., 2021; Navarro-Main et al., 2021; Medeiros et al., 2022). Demyelination, blood–brain barrier impairment, and increased neuroinflammation are revealed in the thalamus of late stage TBI, which is accompanied by the appearance of sleep spindles and epilepsy^64,65^. Given that the hippocampus is a fundamental region in regulating mood and memory (Tartt et al., 2022) and is particularly vulnerable to brain injury, even to mild TBI (Obenaus et al., 2023), our present study provided evidence that the neurological dysfunction after TBI in mice is associated with the exacerbation of inflammation and oxidative stress in hippocampus, demonstrating that hippocampus would be a crucial brain region for preventing and ameliorating the secondary neurological dysfunctions at the late stage of TBI. Future studies are still needed to explore the link between the damage of neurological functions and the pathophysiological changes in different brain regions in the late stage of TBI.

Different mechanisms of neuronal death have been reported during the acute phase of TBI, including apoptosis, autophagy, necrosis, and ferroptosis (Dong et al., 2018; Liu et al., 2018; Xie et al., 2019; Akamatsu and Hanafy, 2020; Sarkar et al., 2020; Cheng et al., 2023). Abnormal autophagy is detected in the perilesional cortex after 12 weeks following TBI (Ritzel et al., 2022). However, few studies have focused on the pattern of cell death in the ipsilateral hippocampus during chronic phase of TBI. In the present study, we disclosed that apoptosis accounted for the long-term chronic neuronal loss in the ipsilateral hippocampus following TBI, which is consistent with the previous study that elevated cleaved-caspase-3 in the ipsilateral thalamus resulted in chronic myelin pathology following TBI (Glushakov et al., 2018). Further studies on the manner of neuronal impairment in different regions during the late phase of TBI are warranted.

During the early stage of TBI, excessive ROS are generated by granulocytes and macrophages rather than activated or resting microglia, leading to extensive oxidative stress at the lesion site (Abe et al., 2018; Wu et al., 2022). In the chronic phase of TBI, microglia or macrophages contribute more to ROS generation and oxidative stress in the injured brain, which is supported by the increased NADPH oxidase (NOX2) in the microglia or macrophages at 1 year post injury (Loane et al., 2014). Although we did not explore the source of ROS in the late phase of adolescent TBI, the aggravated oxidative stress in the ipsilateral hippocampus may contribute to the neurological dysfunction in middle-aged mice. Our present data is consistent with the previous study that oxidative stress is one of the etiologies of various neurodegenerative diseases (George et al., 2022).

It has been reported that astrocytes and microglia/macrophages play important roles in the initiation and persistence of inflammatory responses following TBI (Mira et al., 2021). Our present study revealed that the persistent reactive astrocytes undergo morphological changes in the ipsilateral hippocampus during the whole observed chronic phase, which is consistent with the previous study that reactive gliosis is maintained for up to 60 days following injury in the CCI model (Villapol et al., 2014). The reactive astrogliosis may secrete various cytokines/chemokines and transform into A1 neurotoxic astrocytes to influence the local inflammatory microenvironment, which is supported by the evidence that astrocytic complement C3 is linked to various neurological diseases (Yun et al., 2018; Clark et al., 2019; Li et al., 2022; Stym-Popper et al., 2023). In line with the intricate spatial morphological structure of reactive astrogliosis post injury, we observed that certain astrocytes in the hippocampal hilus underwent morphological complexity, which might contribute to the neuronal damage in the chronic phase of TBI.

Microglia is recognized as the most important contributor to inflammation following TBI. Reactive microglia are found in multiple brain regions in the early and late stages of TBI (Loane et al., 2014). In our study, M1 subtype of the microglia was demonstrated to be a chronic and possibly lifelong event in the ipsilateral hippocampus post injury, which is also consistent with the previous opinion that microglia maintains a prolonged proinflammatory state after brain trauma (Loane et al., 2014). Some studies have reported that the reactivate microglia and the proinflammatory subtype of microglia may enhance the phagocytosis of degenerative axons and synapsis in mouse models of multiple neurodegenerative diseases (Alawieh et al., 2021; Guo et al., 2022). Though we did not test the phagocytotic capability of reactive microglia in hippocampus, the reactive microglia in the late stage of TBI may partly account for the neurological dysfunction in the late life of TBI mice.

Considering that NRF2 is the most important element for mediating antioxidant and anti-inflammatory response in both human and rodent TBI models (Dong et al., 2018, 2019; Guo et al., 2019; He et al., 2019), our previous study reveals that NRF2 is ubiquitously activated in neurons, astrocytes, microglia, and oligodendrocytes in the early stages of TBI in a spatial and temporal pattern (Dong et al., 2018, 2019; Guo et al., 2019; He et al., 2019). In our present study, persistent NRF2, and its regulated genes, HMOX-1 and NQO-1 were triggered by TBI within 6 mpi. However, it is not sufficient to antagonize the chronic oxidative stress. In addition, NRF2-mediated antioxidative response decreased sharply in the middle-aged mice (12 mpi), which is consistent with the previous studies that downregulation of NRF2 results in increased oxidative stress in aged mice (Zhang et al., 2015; Schmidlin et al., 2019) and neurodegenerative diseases (Osama et al., 2020; Zgorzynska et al., 2021). The lack of NRF2 in the late life of TBI mice may also account for the enhanced inflammation and neural dysfunction, which is supported by the evidence that NRF2 ablation leads to the increase of proinflammatory cytokines and modulation of microglial dynamics (Rojo et al., 2010; Chen et al., 2018). Thus, chronic oxidative stress and neuroinflammation in the hippocampus may be linked with the physical deficiency of NRF2 in the late life of TBI mice, which also needs further investigation.

## Conclusion

Our study describes the dynamic neurological dysfunction in the chronic late phase of TBI. The persistent neuroinflammation and oxidative stress contribute to the neuronal apoptosis and neural dysfunction in the late life of adolescent TBI mice. The aggravated neurological dysfunction in middle-age of the TBI mice may be partly associated with the physical deficiency of NRF2. Our present study provides significant insights into the mechanism underlying the long-term neurological decline post-injury in adolescent individuals and the detrimental effects of TBI in the late life of the victims.

## Data availability statement

The raw data supporting the conclusions of this article will be made available by the authors, without undue reservation.

## Ethics statement

The animal study was approved by Animal Ethics Committee of China Medical University. The study was conducted in accordance with the local legislation and institutional requirements.

## Author contributions

ZC: Writing – original draft. PW: Formal analysis, Writing – review & editing. HC: Methodology, Writing – review & editing. NW: Writing – review & editing. MW: Writing – review & editing. ZiW: Writing – review & editing. ZhW: Writing – review & editing. WD: Writing – review & editing. DG: Writing – review & editing. LW: Writing – review & editing. RZ: Funding acquisition, Project administration, Supervision, Writing – review & editing.
