# Supplementary material for: Adolescent traumatic brain injury leads to incremental neural impairment in middle-aged mice: role of persistent oxidative stress and neuroinflammation
Source: Front Neurosci. 2023 Oct 25;17:1292014. doi: 10.3389/fnins.2023.1292014 (PMC10642192; doi:10.3389/fnins.2023.1292014)
Supplement: Supplementary file 1 [file Table_1.DOCX]

***Supplementary Materials***

Table S1. Information of antibodies used in this study

| **Antibody** | **Host** | **Distributor** | **Cat.No.** | **Dilution** |
| --- | --- | --- | --- | --- |
| **Primary** |  |  |  |  |
| GFAP | Rabbit | Proteintech | 16825-1-AP | 1:400 (IF) |
|  |  |  |  | 1:1000 (WB) |
| C3d | Goat | R&D | AF2655 | 1:200 (IF) |
| NeuN | Rabbit | MilliporeSigma | ABN78 | 1:200 (IF) |
| IBA1 | Goat | Abcam | Ab5076 | 1:200 (IF) |
|  |  |  |  | 1:1000 (WB) |
| IBA1 | Rabbit | Wako | 019-19741 | 1:800 (IF) |
| CD16/32 | Goat | R&D | AF1460 | 1:200 (IF) |
|  |  |  |  | 1:1000 (WB) |
| ARG1 | Rabbit | Proteintech | 16001-1-AP | 1:400 (IF) |
|  |  |  |  | 1:1000 (WB) |
| 4-HNE | Mouse | R&D | MAB3249 | 1:400 (IHC) |
|  |  |  |  | 1:1000 (WB) |
| Cleaved caspase-3 | Rabbit | CST | 9661 | 1:400 (IHC) |
|  |  |  |  | 1:1000 (WB) |
| IL-1β | Rabbit | Absin | abs-115412 | 1:1000 (WB) |
| IL-6 | Rabbit | Proteintech | 21865-1-AP | 1:1000 (WB) |
| TNF-α | Rabbit | Proteintech | 60291-1-lg | 1:1000 (WB) |
| NRF2 | Rabbit | Abcam | Ab62352 | 1:1000 (WB) |
| HO-1 | Rabbit | Proteintech | 10701-1-AP | 1:1000 (WB) |
| NQO-1 | Rabbit | Proteintech | 11451-1-AP | 1:1000 (WB) |
| GCLC | Rabbit | Proteintech | 12601-1-AP | 1:1000 (WB) |
| GCLM | Rabbit | Proteintech | 14241-1-AP | 1:1000 (WB) |
| **Secondary** |  |  |  |  |
| Donkey anti-rabbit IgG, Alexa Fluor 594 | Donkey | Thermo Scientific | A-21207 | 1:400 (IF) |
| Donkey anti-rabbit IgG, Alexa Fluor 488 | Donkey | Thermo Scientific | A-21206 | 1:400 (IF) |
| Donkey anti-Goat IgG, Alexa Fluor 488 | Donkey | Thermo Scientific | A-11055 | 1:400 (IF) |
| Goat anti-mouse IgG | Goat | ZSGB-BIO | ZB-2305 | 1:1000(WB) |
| Goat anti-rabbit IgG | Goat | ZSGB-BIO | ZB-2301 | 1:1000(WB) |
| Rabbit anti- Goat IgG | Rabbit | ZSGB-BIO | ZB-2306 | 1:1000(WB) |
|  |  |  |  |  |

Table S2. The primers used in qRT-PCR

| Gene | Forward | Reverse |
| --- | --- | --- |
| *Il–1β* | ACGGACCCCAAAAGATGAAG | TTCTCCACAGCCACAATGAG |
| *Il–6* | CAAAGCCAGAGTCCTTCAGAG | GTCCTTAGCCACTCCTTCTG |
| *Tnf–α* | CTTCTGTCTACTGAACTTCGGG | CAGGCTTGTCACTCGAATTTTG |
| *Nrf2* | CGAGATATACGCAGGAGAGGTAAGA | GCTCGACAATGTTCTCCAGCTT |
| *Hmox-1* | ACAGAGGAACACAAAGACCAG | GTGTCTGGGATGAGCTAGTG |
| *Nqo-1* | TGAAGAAGAGAGGATGGGAGG | GATGACTCGGAAGGATACTGAAAG |
| *Gapdh* | CTTTGTCAAGCTCATTTCCTGG | TCTTGCTCAGTGTCCTTGC |

Table S3. The proportion of TBI mice selected for subsequent morphological and molecular biological detection.

|  | Behavior tests | Parameters | Mpi（percentage） | | | |
| --- | --- | --- | --- | --- | --- | --- |
|  |  |  | 1 | 3 | 6 | 12 |
| Depression | SPT | Sucrose consumption | 70% | 70% | 70% | 80% |
|  | FST | Immobility time | 70% | 65% | 75% | 95% |
| Anxiety | EPM | % Time in open arms | 90% | 90% | 85% | 95% |
|  |  | number of entries in open arm | 90% | 90% | 90% | 90% |
| Memory deficit | MWM | Path length in target quadrant | 80% | 80% | 85% | 90% |
|  |  | Time in target quadrant | 80% | 90% | 85% | 100% |
|  |  | Number of platform quadrant | 75% | 70% | 95% | 100% |
|  |  | Number of platform | 70% | 90% | 85% | 100% |
| Number of mice selected |  |  | 12 | 12 | 13 | 15 |


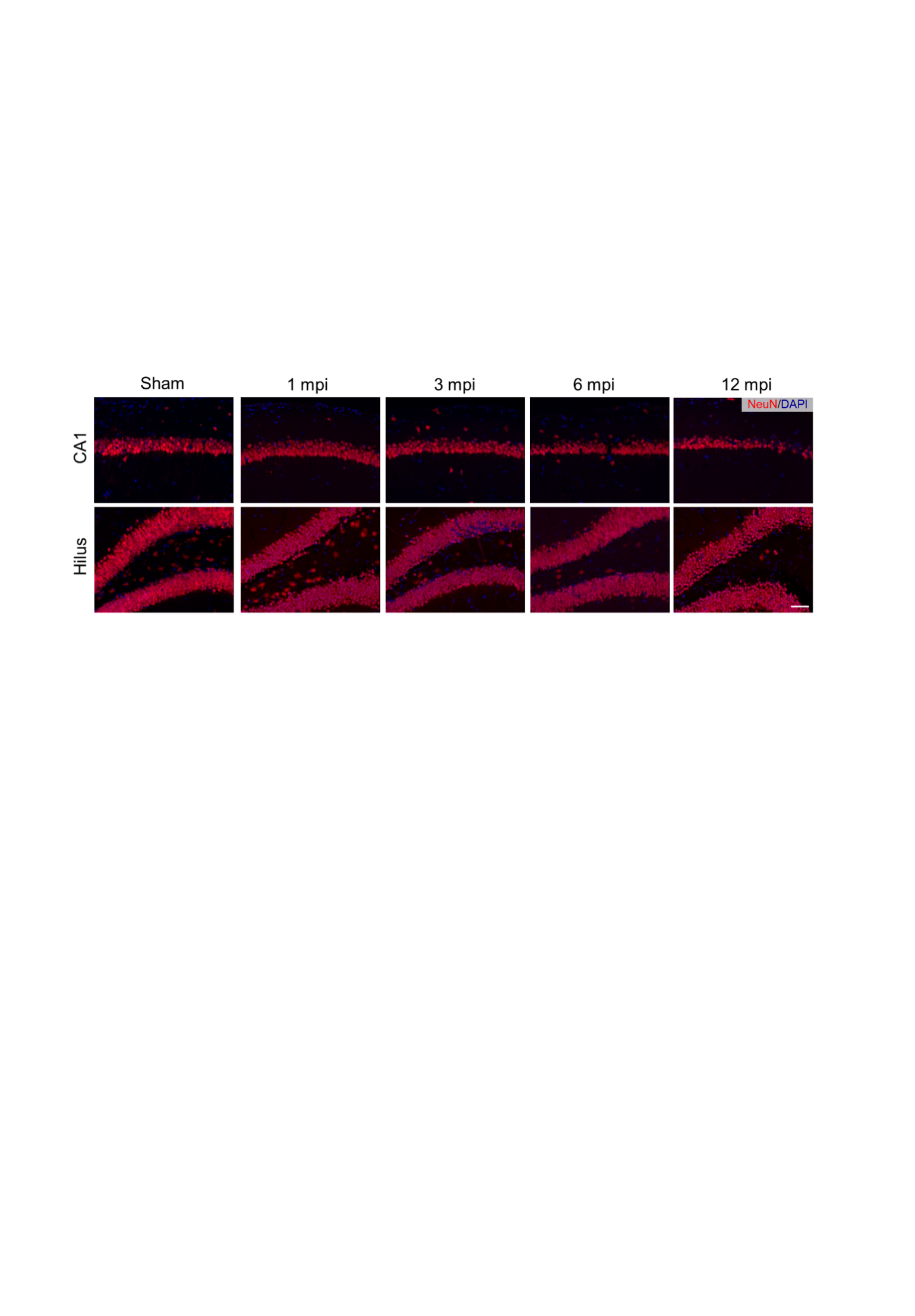


Figure S1. Temporal changes in the number of neurons in hippocampal CA1 and hilus regions after TBI. Representative images of NeuN (red) immunofluorescent staining of ipsilateral hippocampal CA1 at 1, 3, 6 and 12 mpi (upper panel) and hilus (lower panel); Scale bar, 50 μm.


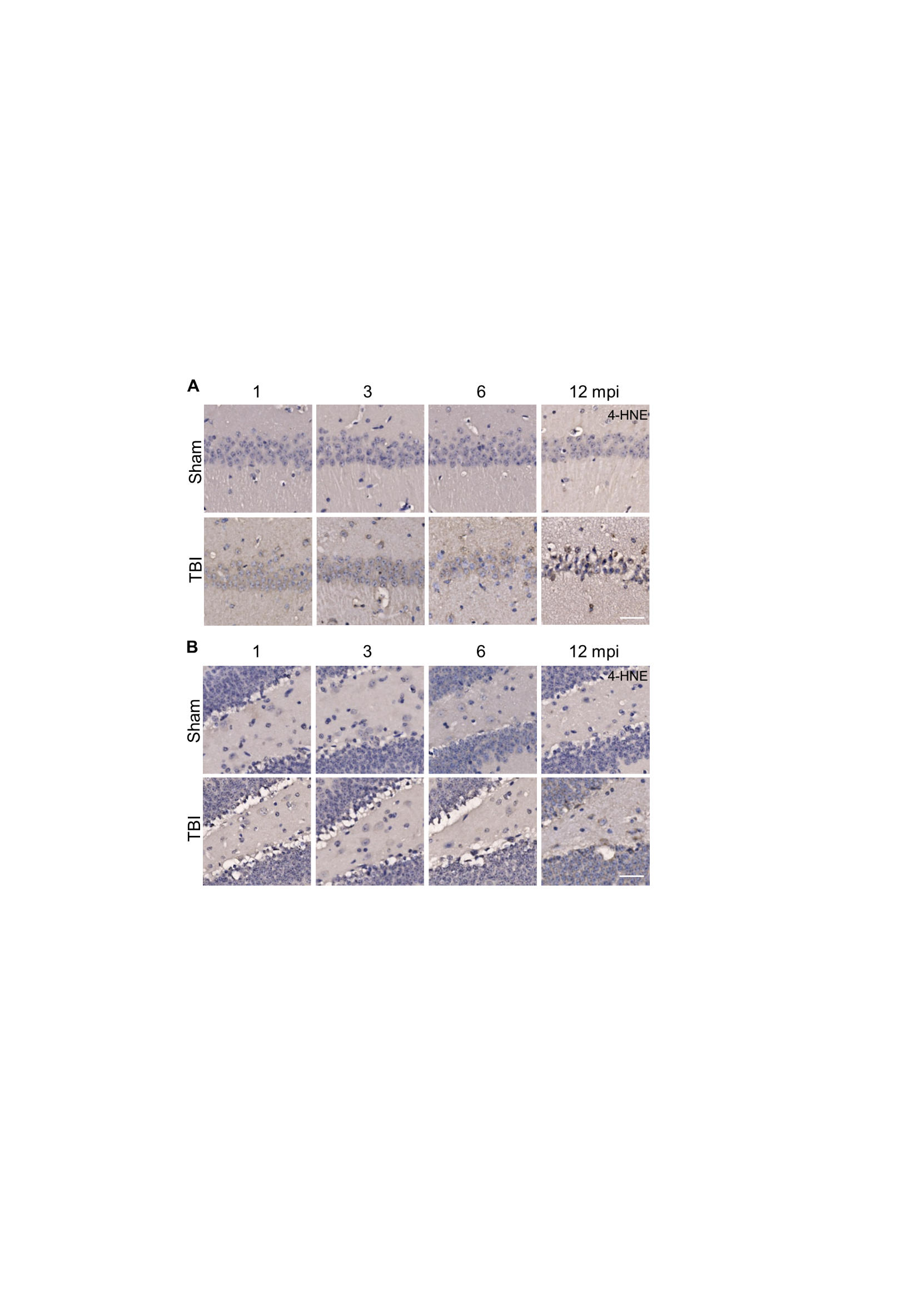


Figure S2. Representative images of 4-HNE immunohistochemical staining of hippocampal CA1 and hilus region at 1, 3, 6 and 12 mpi. (A) Representative images of 4-HNE immunohistochemical staining of hippocampal CA1. (B) Representative images of 4-HNE immunohistochemical staining of hippocampal hilus; Scale bar, 20 μm.


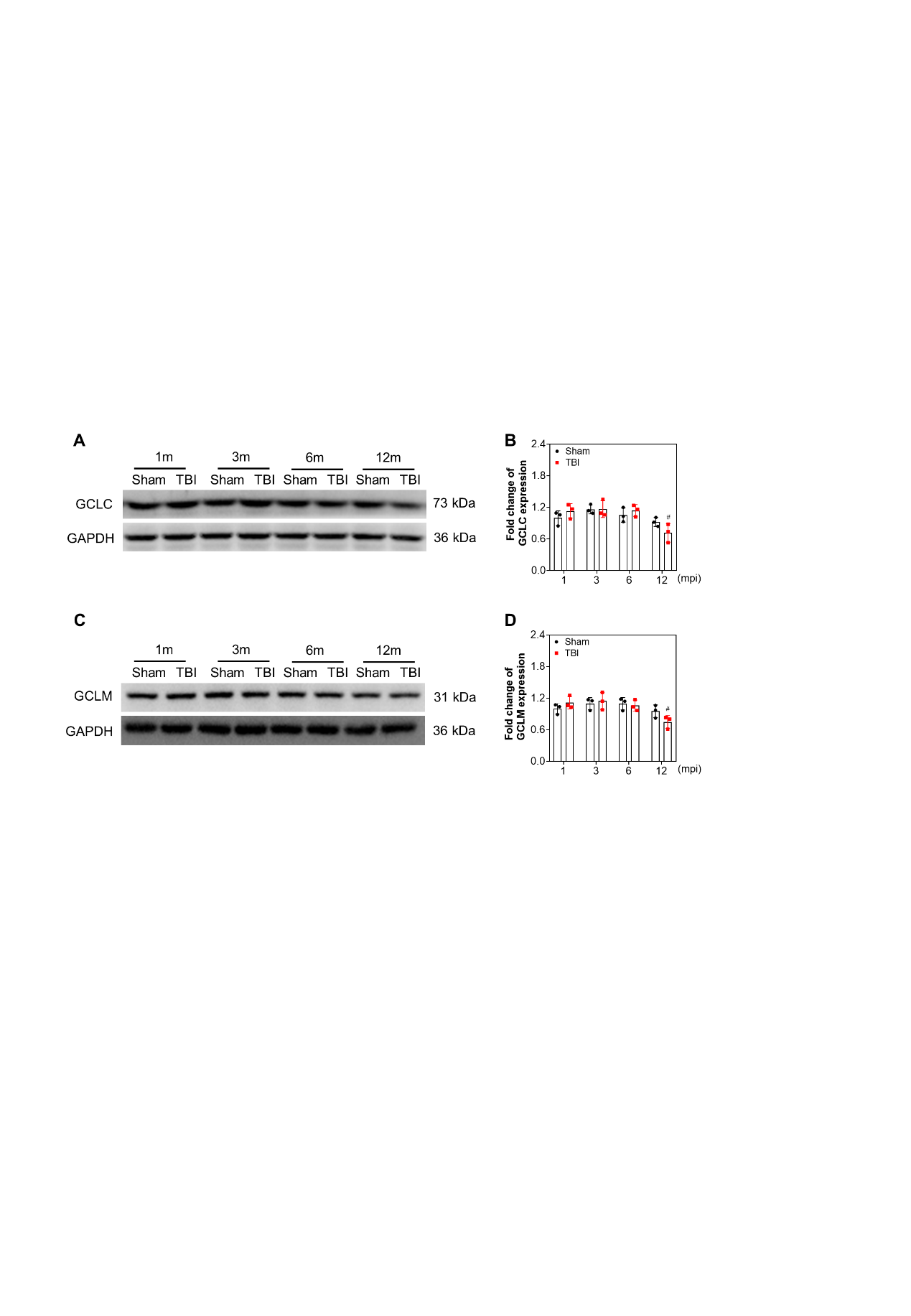


Figure S3. The expression of GCLC and GCLM in hippocampus at the chronic phase of TBI. (A, B) Representative immunoblots and relative densities of GCLC of the ipsilateral hippocampus, n = 3. (C, D) Representative immunoblots and relative densities of GCLM of the ipsilateral hippocampus, n = 3. * *p* < 0.05 compared age-matched sham (Mann-Whitney U Test); # *p* < 0.05 compared with the TBI mice of previous adjacent group (Tukey's post hoc test).


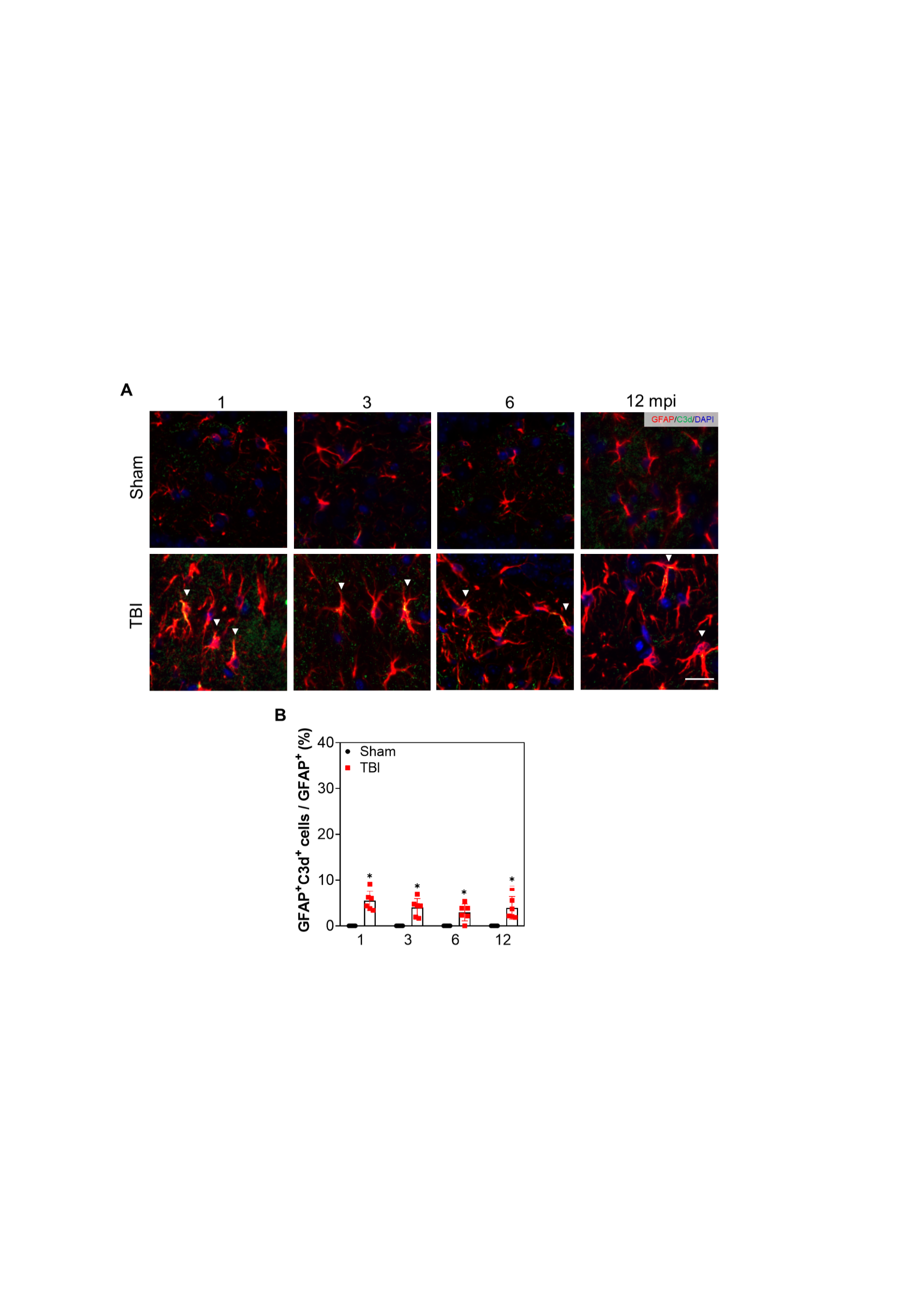


Figure S4. Percentage of C3d positive astrocytes in hippocampal hilus at chronic phase of TBI. (A) Representative images of immunofluorescent double staining of GFAP (red) and C3d (green) in ipsilateral hippocampal hilus. (B) Percentage of GFAP and C3d double positive cells in each group. n = 6; **P* < 0.05 compared age-matched sham (Mann-Whitney U Test). White arrow, GFAP and C3d double labeled cells; Scale bar, 20μm.
